# Transthoracic echocardiographic and artificial intelligence-enabled electrocardiography predictors of atrial arrhythmia recurrence after surgical ablation

**DOI:** 10.1016/j.hroo.2025.11.001

**Published:** 2025-11-07

**Authors:** Dylan Goings, Ikram U. Haq, Arman Arghami, Zachi Attia, Gabor Bagameri, Michael Brandt, Freddy Del-Carpio Munoz, Paul A. Friedman, Kimberly A. Holst, Peter A. Noseworthy, Konstantinos C. Siontis, Alan Sugrue, Ammar M. Killu

**Affiliations:** 1 Department of Internal Medicine Mayo Clinic, Rochester, Minnesota; 2 Department of Cardiovascular Medicine, Mayo Clinic, Rochester, Minnesota

**Keywords:** Atrial fibrillation, Surgical ablation, Artificial intelligence, Echocardiography, Risk prediction, Arrhythmia recurrence, Electrocardiography, Machine learning, Prognostic modeling, Left atrial remodeling

## Abstract

**Background:**

Recurrence of atrial fibrillation (AF)/flutter (AFl) after surgical ablation remains difficult to predict. Integration of novel biomarkers may enhance risk stratification.

**Objective:**

This study aimed to assess whether combining preoperative transthoracic echocardiography (TTE) and artificial intelligence-enabled electrocardiography (AI-ECG) scores improves the prediction of AF/AFl recurrence after surgical ablation.

**Methods:**

We retrospectively analyzed 1696 patients who underwent surgical AF/AFl ablation from 2006 to 2025 with available preoperative TTE and ECG and postblanking (90-day) ECG follow-up. Clinical, TTE, and AI-ECG variables (AF probability, ECG-estimated age, heart failure with preserved ejection fraction, left ventricular dysfunction, and aortic stenosis scores) were assessed. Cox proportional hazards and random survival forest models (80:20 train-test split) identified predictors of recurrence.

**Results:**

Among 1696 patients (mean age 67.3 ± 10.2 years; 61.7% male), 949 (56%) had AF/AFl recurrence over a median 3.14-year follow-up. Patients with recurrence had larger left atrial area (30.4 vs 24.5 cm^2^), elevated mitral E-wave velocity (1.015 vs 0.896 m/s), and adverse AI-ECG biomarkers for AF probability, ECG-estimated age, heart failure with preserved ejection fraction, left ventricular dysfunction, and aortic stenosis (all *P* < .001). In multivariable analysis, independent predictors of recurrence included higher ECG-AF probability (*P* < .0001), older ECG-estimated age (*P* = .0002), left atrial area (*P* = .046), body mass index (*P* = .036), and diastolic blood pressure (hazard ratio 1.008/mm Hg; *P* = .010). The final Cox model achieved a concordance index of ∼0.67 and a 3-year Brier score of 0.21, with 3-year freedom-from-arrhythmia rates of ∼85% vs ∼43% for the lowest- vs highest-risk quartiles. Random survival forest modeling yielded a slightly higher concordance index (∼0.69).

**Conclusion:**

Preoperative AI-ECG biomarkers (AF probability, age discordance) and TTE markers of atrial remodeling independently predicted AF/AFl recurrence after surgical AF/AFl ablation. Integration of these metrics improved risk stratification.

**Key Findings**

▪ Independent predictors: larger left atrial size, higher artificial intelligence (AI)-enabled electrocardiography (ECG) atrial fibrillation (AF) probability, and older ECG-estimated age were independent predictors of postsurgical AF/atrial flutter recurrence, underscoring the prognostic value of both structural and electrical markers of atrial remodeling.
▪ Model performance: the multimodal Cox model showed moderate discrimination (C-index ∼0.67) but excellent calibration (3-year Brier score 0.21), with 3-year arrhythmia-free survival of 85% vs 43% across lowest- and highest-risk quartiles (log-rank χ^2^ = 304; *P* < 10^-60^). These findings indicate strong calibration and clinically meaningful stratification despite modest global ranking performance.
▪ Model robustness: predictive performance and key associations were consistent across surgical eras and missing-data sensitivity analyses, supporting model stability and generalizability.
▪ Clinical implications: this model enables risk-adapted rhythm surveillance, identifying patients at higher risk of recurrence who may benefit from intensified follow-up or risk-factor modification while supporting routine care in lower-risk groups.
▪ Novelty and future directions: this study is the first, to the best of our knowledge, to evaluate the prognostic utility of AI-enabled ECG biomarkers in a surgical AF ablation population. Future research should focus on prospective validation and incorporation of dynamic or multimodal AI-derived imaging features to further refine prediction accuracy.

## Introduction

Atrial fibrillation (AF) and flutter (AFl) recurrence remains a significant challenge after surgical ablation. Although the Cox-Maze IV procedure achieves high acute success rates (80%–90% freedom from AF at 1 year),^1^ up to 20% of patients experience late recurrence of atrial arrhythmias.^2^ Traditional predictors of recurrence include left atrial (LA) enlargement, AF duration, hypertension, and obesity.^3^ These reflect underlying structural and electrical remodeling.^4^ Among these, LA size has emerged as the most consistent echocardiographic predictor of procedural failure in surgical cohorts.^5^ However, conventional clinical and imaging parameters lack sufficient discriminatory power to reliably stratify individual risk, underscoring the need for advanced tools to optimize postoperative surveillance and personalized management.

Recent advances in artificial intelligence-enabled electrocardiography (AI-ECG) have demonstrated remarkable potential to identify latent cardiovascular pathology from sinus rhythm tracings.^6^ Deep-learning algorithms can detect subtle electrical signatures of atrial myopathy, ventricular dysfunction, and even discrepancies between chronological and biological age. Beyond arrhythmia-specific applications, AI-ECG algorithms can now identify occult structural diseases such as heart failure with preserved ejection fraction (HFpEF) and valvular heart disease, further highlighting their utility in risk stratification.^7^^,^^8^ These findings raise a critical question: can AI-ECG biomarkers augment traditional echocardiographic indices to refine prognostication in surgical AF/AFl ablation?

In this multicenter retrospective cohort study, we aimed to evaluate the synergistic prognostic value of preprocedural transthoracic echocardiography (TTE) and validated AI-ECG risk scores. We hypothesized that integrating AI-derived markers, including ECG-predicted AF probability, ECG-estimated age, and scores indicating HFpEF, left ventricular (LV) dysfunction, and aortic stenosis (AS), with conventional LA remodeling metrics would improve long-term prediction of atrial arrhythmia recurrence.

## Methods

### Study population and data collection

We used an electronic database maintained by Mayo Clinic (using natural language processing for data retrieval) to identify patients undergoing surgical AF/AFl ablation between 2006 and 2025 at Mayo Clinic across 3 sites: Rochester, MN; Scottsdale, AZ; and Jacksonville, FL. Procedures were classified as standalone or concomitant (surgical ablation performed alongside other cardiac surgery). The inclusion criteria required a comprehensive TTE within 6 months before the index procedure and at least 1 postoperative 12-lead ECG ≥1 year after surgery to ensure adequate follow-up. Patients were excluded if they lacked follow-up ECG data or had incomplete key baseline data.

Baseline demographics (age, sex, body mass index [BMI]), clinical comorbidities (hypertension, heart failure, diabetes), AF subtype (paroxysmal vs persistent), and surgical context (standalone vs concomitant surgical ablation) were extracted from the electronic health records. Preoperative TTE parameters were obtained from structured reports, including LA volume index (LAVI) (biplane Simpson’s method), LV ejection fraction (LVEF) (biplane Simpson’s method), LV mass, LV end-diastolic diameter, and diastolic function indices: mitral inflow E/A ratio, septal/lateral e’ velocities, and E/e’ ratio.

From the most recent preoperative ECG (median 2.56 days before surgery; interquartile range [IQR] 0.67–10.64), we obtained the following validated AI-ECG risk scores:

• ECG-AF: probability of underlying AF (trained on 454,789 ECGs; area under the curve [AUC] 0.87^1^ in external validation)^6^
• ECG-Age: predicted biological age derived from 12-lead ECG features (trained on 499,727 patients; AUC 0.94 for detection of age ≥40)^9^
• ECG-HFpEF: probability of HFpEF (trained on 98,736 patients; AUC 0.911 for detecting increased filling pressure)^7^
• ECG-AS: probability of moderate/severe AS (trained on 129,788 patients; AUC 0.85 in external validation)^8^
• ECG-low EF: probability of LVEF of 35% or below (trained on 44,959 patients; AUC 0.93 in external validation)^10^

All AI-ECG scores were generated using previously validated deep-learning models. None of the ECGs included in this surgical ablation cohort were part of the original training or validation datasets for these algorithms, thereby avoiding model-sample overlap and potential overfitting.

### Ethics statement

This retrospective study was approved by the Mayo Clinic Institutional Review Board. All patients had previously provided written authorization for the use of their medical records for research in accordance with institutional policy and the principles of the Declaration of Helsinki. No additional patient contact or intervention occurred for this study.

### Outcomes

The primary outcome was time to the first documented AF/AFl recurrence on any follow-up 12-lead ECG after a 90-day postoperative blanking period. Patients were classified into a no recurrence group (no AF/AFl recurrence after the blanking period) or a recurrence group (AF/AFl recurrence observed during follow-up). Patients were censored at the time of the last clinical follow-up, death, or loss to contact.

### Statistical analysis

The key objectives of our analysis were to develop a multivariable Cox proportional hazards model to identify preoperative predictors of recurrence, assess its discrimination and calibration, and compare its performance with a machine-learning survival model (random survival forest [RSF]). All analyses were performed using Python (v3.9) and R (v4.1). Each clinical, echocardiographic, and AI-ECG variable was first tested in a univariate Cox proportional hazards model for association with recurrence. Continuous variables were modeled linearly unless otherwise specified. Variables with *P* < .05 on univariate analysis were considered statistically significant and retained as candidates for multivariable modeling. Cases with missing data were handled by listwise deletion in the univariate analyses.

For multivariable modeling, we used a hybrid variable selection approach combining data-driven methods and clinical judgment. Significant univariate predictors were used to fit numerous candidate Cox models using random subsets of 5–15 predictors. Missing data were handled using multiple imputation by chained equations, with 5 imputed datasets generated for each model (with multiple imputation by chained equations to account for missing data in each model). We tracked each variable’s frequency of significance (*P* < .05) in models with good discrimination (concordance index [C-index] >0.55) to identify robust predictors. Variables that consistently emerged as significant and that were clinically plausible were prioritized for inclusion in the final multivariable model.

The final multivariable Cox model included 14 predictors: ECG-AF score, ECG-age, ECG-AS score, ECG-HFpEF score, diastolic blood pressure, BMI, LA area (apical 4-chamber), age at surgery, mitral E-wave peak velocity, pulmonary valve peak systolic velocity, LV end-diastolic dimension, heart rate (at time of stroke volume measurement), LVEF, and LV mass. Of note, the LA area was retained in the multivariable model over LAVI owing to its strong univariate performance, consistent availability across the cohort, and lower dependence on assumptions such as accurate body surface area or high-quality apical 2-chamber imaging. Model performance was assessed by Harrell’s C-index. The proportional hazards assumption was verified for all covariates using Schoenfeld residuals.

We evaluated model calibration by comparing predicted with observed recurrence-free survival at 3 years. Patients were stratified into quartiles of predicted risk, and Kaplan–Meier survival curves were plotted for each quartile. The observed 3-year freedom-from-recurrence rates in each risk quartile were compared with the model’s predictions.

To explore potential nonlinear interactions among predictors, we also trained an RSF model. The RSF was implemented with the scikit-survival library, using 300 trees and a minimum of 10 samples per split, and was trained on 80% of the cohort (with the remaining 20% used for testing). Missing data in the RSF model were handled using the same multiple imputation strategy as in the Cox model. Model performance was evaluated by the C-index on the test set. Permutation-based feature importance was computed across imputed datasets, and the top predictive variables were compared with those in the Cox model to assess consistency across modeling approaches.

## Results

### Patient characteristics

The study cohort comprised 1696 patients undergoing surgical AF/AFl ablation (mean age 67.3 ± 10.2 years; 61.7% male), with a median follow-up of 3.14 years (IQR 1.8–5.1). Among these, 949 patients (56%) developed recurrent AF/AFl after the 90-day blanking period (recurrence group), whereas 747 (44%) remained arrhythmia-free at last follow-up (no recurrence group). The median time to the first recurrence in the recurrence group was 38.5 months (IQR 18.2–62.7).

Longitudinal rhythm follow-up was available for all 1771 patients. After exclusion of ECGs within the 90-day post-Maze blanking period, every patient had at least 1 subsequent ECG (median 7 [IQR 3–15] postblanking ECGs per patient). The first postblanking ECG occurred a median of approximately 3 months after surgery, and the median ECG acquisition rate beyond the blanking period was 1.4 per patient-year (IQR 0.8–2.5). Across the cohort, 20% of ECGs occurred within 6–12 months, 25% between 12 and 24 months, and the remainder after 24 months, indicating consistent long-term rhythm surveillance.

Baseline demographics, clinical characteristics, and imaging parameters by group are presented in Table 1. Patients in the recurrence group were older (70.1 ± 9.8 vs 63.4 ± 9.5 years; *P* < .001), more often male (64.3% vs 58.4%; *P* = .007), and more frequently obese (BMI ≥30 kg/m^2^ in 42.1% vs 34.8%; *P* = .003) than the no recurrence group. Comorbidities such as hypertension (82.6% vs 74.2%; *P* < .001) and persistent AF (68.9% vs 51.2%; *P* < .001) were also more prevalent in the recurrence group. Because subtype documentation was inconsistently reported across eras and sometimes changed longitudinally, AF type was not included in multivariable modeling. Echocardiographic markers differed between groups: patients with recurrence had larger LAVIs (54.8 vs 50.0 mL/m^2^) and more pronounced diastolic dysfunction, evidenced by higher E/e’ ratios (16.3 vs 13.4) and lower septal e’ velocities (0.071 vs 0.078 cm/s). LVEF was preserved and similar between groups (58% ± 10% vs 57% ± 11%; *P* = .21).

**Table 1:** Baseline characteristics

Characteristic	Recurrence group	No recurrence group
Total patients (% male)	949 (64.3)	747 (58.4)
Time to follow-up/recurrence (mo)	38.5 ± 37.1	67.6 ± 46.3
Age at ablation (y)	69.20	63.36
Body mass index	29.5 ± 6.3	28.4 ± 6.0
LVEF (%)	55.0 ± 10.3	55.9 ± 11.1
LA end-systolic length (2D averaged view)	65.89	58.49
Mitral valve E/e’ ratio	16.3 ± 14.2	13.4 ± 7.8

2D = 2-dimensional; LA = left atrial; LVEF = left ventricular ejection fraction.

### Univariate predictors of recurrence

Univariate Cox analysis identified 73 of 155 tested variables as significant predictors of AF/AFl recurrence (*P* < .05) (see Table 2). A comprehensive list of all univariate results is presented in Supplemental Table 1. Traditional echocardiographic markers of adverse atrial remodeling and diastolic dysfunction showed particularly strong associations. For example, each 1 cm^2^ increase in LA area (apical 4-chamber view) conferred a hazard ratio (HR) of 1.052 (*P* = 2.8×10^-23^); patients in the recurrence group had a mean LA area of 30.4 cm^2^ compared with 24.5 cm^2^ in those without recurrence. Mitral inflow E-wave velocity of ≥1.0 m/s (vs <0.9 m/s) was associated with a nearly 2-fold hazard (HR 1.76; 95% confidence interval [CI] 1.58–1.97; *P* < .001). Elevated filling pressures, quantified by an E/e’ ratio, likewise predicted recurrence (HR 1.018; 95% CI 1.016–1.021).

**Table 2:** Univariate predictors of AF/AFl recurrence after surgical ablation

Predictor	Hazard ratio	95% CI (lower)	95% CI (upper)	*P* value
ECG-AF∗	1.014	1.012	1.016	1.9 × 10^-48^
ECG-age	1.052	1.043	1.060	6.6 × 10^-35^
ECG-HFpEF∗	1.301	1.232	1.375	3.6 × 10^-21^
ECG-AS∗	1.009	1.007	1.011	2.9 × 10^-14^
ECG-low EF∗	1.006	1.005	1.008	6.9 × 10^-6^
LA end-systolic length (2D 4-chamber)	1.056	1.046	1.066	1.3 × 10^-24^
LA area (2D apical 4-chamber)	1.052	1.042	1.063	2.8 × 10^-23^
Age at Maze	1.024	1.019	1.029	5.4 × 10^-20^
Mitral valve E-wave peak velocity	1.761	1.504	2.063	9.5 × 10^-12^
LA end-systolic diameter (2D)	1.085	1.051	1.120	3.0 × 10^-9^
LA systolic volume (2D)	1.017	1.012	1.023	1.8 × 10^-8^
Mean arterial pressure	1.020	1.014	1.027	2.2 × 10^-8^
Diastolic blood pressure	1.022	1.014	1.030	1.4 × 10^-7^
Body mass index	1.026	1.013	1.038	3.1 × 10^-5^
LA diameter (2D AP view)	1.175	1.101	1.253	3.4 × 10^-5^

2D = 2-dimensional; AF = atrial fibrillation; AFl = atrial flutter; AP = anteroposterior; AS = aortic stenosis; CI = confidence interval; ECG = electrocardiography; EF = ejection fraction; HFpEF = heart failure with preserved ejection fraction; LA = left atrial.

∗ Hazard ratios for ECG-AF, ECG-AS, and ECG-low EF are expressed per 0.01-unit (1%) increase in the model output to enhance interpretability. The ECG-HFpEF score is reported per 1-unit increase on its native 0–3 scale. All other covariates are presented per their original measurement units.

For interpretability, all AI-ECG outputs ranging from 0 to 1 were expressed as HRs per 0.01-unit (1%) increase in model probability. AI-ECG risk scores emerged as significant predictors of procedural failure. The ECG-AF probability score showed the strongest univariate association (HR 1.014; 95% CI 1.012–1.016; *P* < .001 per 0.01-unit [1%] increase), followed by the ECG-age (HR 1.052; 95% CI 1.043–1.060; *P* < .001) and the ECG-HFpEF score (HR 1.301; 95% CI 1.232–1.375; *P* < .001 per 1-unit increase on a 0–3 scale). The ECG-AS and ECG-low EF scores were also associated with recurrence (HR 1.009 and 1.006 per 0.01-unit increase, respectively; both *P* < .001). Notably, the AI-derived ECG-age outperformed chronological age: each 1-year increment in ECG-age increased risk by 5.2%, whereas each additional year of chronological age conferred only a 2.4% increase in risk (HR 1.024; 95% CI 1.020–1.028; *P* < .001). Clinical variables such as obesity (per point in BMI, HR 1.026; 95% CI 1.019–1.032; *P* < .001) and elevated diastolic blood pressure (HR 1.02; 95% CI 1.012–1.018; *P* < .001) were also significant, although with smaller effect sizes.

### Multivariable Cox model

To identify independent predictors of recurrence, we developed a multivariable Cox proportional hazards model using 15 candidate variables chosen a priori based on their univariate significance (*P* < .05) and clinical relevance. These included 5 AI-ECG scores (ECG-AF probability, ECG-age, ECG-AS, ECG-HFpEF, ECG-low EF) and 10 clinical/echocardiographic parameters (LA area, mitral E-wave velocity, diastolic blood pressure, BMI, LV end-diastolic dimension, LV mass, LVEF, pulmonary valve systolic velocity, heart rate, and chronological age).

The final model is presented in Table 3. Again, AI-ECG outputs ranging from 0 to 1 were expressed as HRs per 0.01-unit (1%) increase in model probability. After rescaling for interpretability, the ECG-AF probability score demonstrated the strongest adjusted association with recurrence (HR 1.009; 95% CI 1.007–1.011; *P* < .0001 per 0.01-unit increase), followed by the AI-derived ECG-age (HR 1.020; 95% CI 1.010–1.030; *P* = .00023). Chronological age was not an independent predictor once ECG-age was included (HR 1.005; 95% CI 0.998–1.013; *P* = .152), suggesting that ECG-estimated age captures risk attributes beyond chronological aging. Diastolic blood pressure (HR 1.008; 95% CI 1.002–1.014; *P* = .010) also remained independently predictive in the multivariable model. Other variables in the model, such as LA area, mitral E-wave velocity, BMI, ECG-AS, pulmonary valve velocity, heart rate, LVEF, LV mass, and age at surgery, did not individually reach statistical significance (*P* > .05) after adjustment for the stronger predictors. However, they were retained to ensure the model was well-calibrated and represented key clinical domains. The model demonstrated moderate discrimination (C-index 0.67) and excellent calibration (3-year Brier score 0.21), with distinct risk separation across quartiles (3-year arrhythmia-free survival 43% vs 85%; log-rank *P* < 10^-60^).

**Table 3:** Multivariable Cox proportional hazards model identifying independent predictors of AF/AFl recurrence

Predictor	Hazard ratio	95% CI (lower)	95% CI (upper)	*P* value
ECG-AF∗	1.0091	1.00701	1.01111	<1 × 10^-300^
ECG-age (per y)	1.01992	1.00959	1.03035	.00023
ECG-AS∗	1.00184	0.99852	1.00511	.27845
ECG-HFpEF∗	1.06811	0.99487	1.14674	.07568
ECG-low EF∗	0.99829	0.99503	1.00179	.30047
Diastolic blood pressure (per mm Hg)	1.00787	1.00194	1.01384	.00954
Body mass index (per kg/m^2^)	1.01326	1.00106	1.02561	.03579
LA area by apical 4-chamber view (per cm^2^)	1.00907	1.00022	1.01799	.04560
Age at ablation (per y)	1.00534	0.99821	1.01252	.15186
Mitral E-wave peak velocity (per 0.1 m/s)	1.16284	0.93633	1.44415	.18531
Pulmonary valve systolic peak velocity	1.03170	0.87248	1.21997	.72094
LV end-diastolic internal diameter (per mm)	1.00212	0.98928	1.01514	.75438
Heart rate at SV acquisition (per bpm)	1.00059	0.99644	1.00476	.78012
LVEF (per %)	0.99450	0.98640	1.00267	.19013
LV mass (per gram)	1.00002	0.99891	1.00114	.96783

AF = atrial fibrillation; AFl = atrial flutter; bpm = beats per minute; AS = aortic stenosis; CI = confidence interval; ECG = electrocardiography; EF = ejection fraction; HFpEF = heart failure with preserved ejection fraction; LA = left atrial; LV = left ventricular; LVEF = left ventricular ejection fraction; CV = stroke volume.

∗ Hazard ratios for ECG-AF, ECG-AS, and ECG-low EF are expressed per 0.01-unit (1%) increase in the model output to enhance interpretability. The ECG-HFpEF score is reported per 1-unit increase on its native 0–3 scale. All other covariates are presented per their original measurement units.

**Figure 1:**
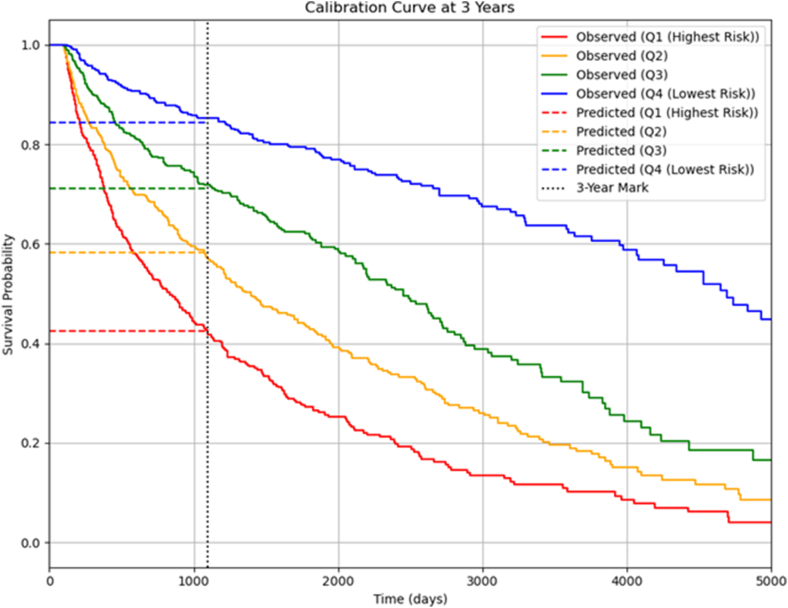
Kaplan–Meier curves for freedom from atrial fibrillation/flutter postsurgical ablation stratified into quartiles of predicted risk according to the Cox model developed.

**Figure 2:**
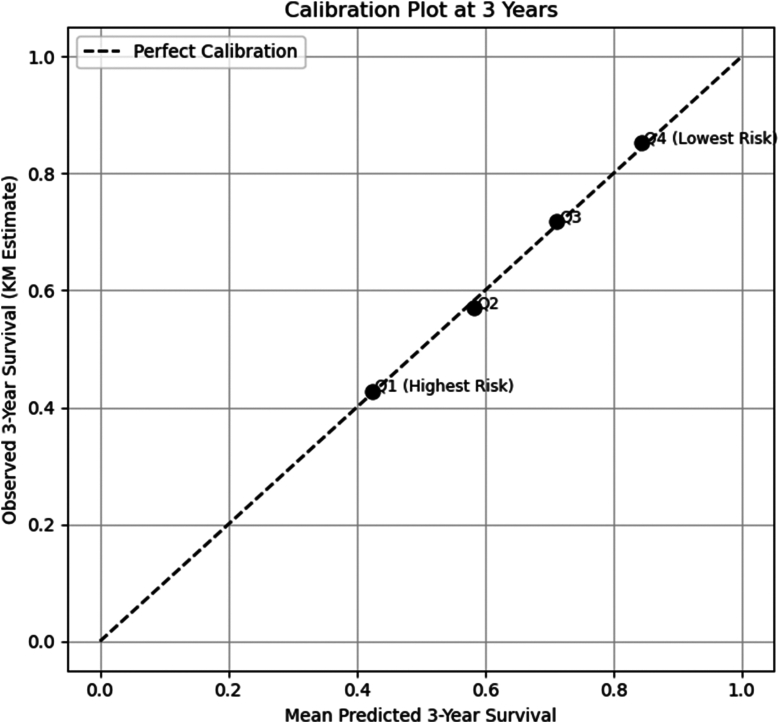
Calibration of the Cox model for predicting atrial fibrillation/flutter recurrence at 3 years

Kaplan–Meier curves stratified by risk quartile are presented in Figure 1. The highest-risk quartile had an estimated 3-year recurrence-free survival of 42.6% (95% CI 37.6–47.3) vs 85.2% (95% CI 81.3–88.3%) in the lowest-risk quartile (log-rank *P* = 1.230 × 10^-65^). Observed 3-year AF/AFL-free survival aligned closely with model predictions across quartiles, as shown in Figure 2.

### Era-stratified and missing-data sensitivity analysis

To assess potential temporal heterogeneity, patients were grouped by surgical era: era 1 (2006–2011), era 2 (2012–2017), and era 3 (2018–2025). In the unadjusted Kaplan–Meier analysis, recurrence rates differed significantly across eras (log-rank *P* = .015). When the full multivariable Cox model was repeated with era included as covariates, era 2 (*P* = .062) and era 3 (*P* = .088) each demonstrated nonsignificant trends toward lower recurrence risk relative to era 1. Inclusion of era did not materially change the strength or direction of the main predictors, including ECG-AF probability (*P* = .002), ECG-age (*P* < .001), ECG-HFpEF (*P* = .036), diastolic blood pressure (*P* = .003), and age at Maze (*P* = .009) (Supplemental Table 2). Model discrimination remained stable (C-index = 0.65).

A complete-case sensitivity analysis was then performed among 565 patients with available LA area measurements. Results were directionally consistent with the multiply imputed main model, with ECG-age and LA area remaining the strongest predictors of arrhythmia recurrence. In the complete-case model, the LA area demonstrated a stronger association (HR 1.031; 95% CI 1.015–1.047; *P* < .001 per cm^2^), whereas ECG-age (HR 1.036; 95% CI 1.025–1.047; *P* < .001 per year) and BMI (HR 1.023; 95% CI 1.002–1.045; *P* = .033) also remained significant. Other covariates preserved direction and relative magnitude, and overall model discrimination was stable (C-index = 0.69 vs 0.65 in the imputed model) (Supplemental Table 3).

### RSF analysis

To explore nonlinear relationships among predictors, we trained an RSF model using the same 15 predictor variables as the Cox model. The RSF achieved marginally higher discrimination, with a test-set C-index of 0.685 vs 0.674 for the Cox model. To evaluate each modality’s contribution, we also developed an AI-ECG–only model and a TTE-only model, thereby directly assessing the added value of multimodal integration. The combined model modestly outperformed both single-modality models (supporting the benefit of integration), as presented in Table 4.

**Table 4:** Model discrimination (C-index) for combined vs single-modality models

C-index	Cox model	Random survival forests
Combined	0.674	0.685
ECG only	0.663	0.673
TTE only	0.574	0.564

C-index = concordance index; ECG = electrocardiography; TTE = transthoracic echocardiography.

However, the RSF’s survival predictions were less straightforward to interpret or calibrate. The RSF’s top variables (by mean permutation importance) were similar to those seen in the Cox model: ECG-AF probability, ECG-age, age at surgery, ECG-HFpEF, and diastolic blood pressure were among the leading contributors to the RSF decisions. This concordance reinforces the relevance of these predictors. Overall, although the machine-learning model hints at potential gains in predictive accuracy with more complex interactions, the improvement over the simpler Cox model was relatively small in our dataset.

## Discussion

The primary findings in this large multicenter cohort of patients undergoing surgical AF/AFl ablation were:

1. Preoperative TTE markers of atrial remodeling (eg, larger LA size) and AI-ECG biomarkers (eg, high AF probability score, older ECG-estimated age) were independent predictors of AF/AFl recurrence after surgical ablation. Although the ECG-AF score showed a strong effect, other predictors had modest effect sizes, and overall model discrimination was moderate (C-index ∼0.67).
2. Integrating AI-ECG with echocardiographic metrics modestly improved risk stratification. The final Cox model stratified patients into distinct risk quartiles with ∼85% 3-year arrhythmia-free survival in the lowest-risk group vs ∼43% in the highest-risk group. Despite moderate discrimination (C-index ∼0.67), the model achieved a 3-year Brier score of 0.21, reflecting excellent calibration, and demonstrated highly significant survival separation across predicted risk quartiles (log-rank χ^2^ = 304; *P* < 10^-60^). Together, these results indicate accurate group-level calibration and strong clinical stratification even if global patient-level ranking was modest.
3. A nonlinear RSF model achieved slightly higher discrimination (C-index ∼0.69) but identified similar top predictors (ECG-AF score, ECG-age, diastolic blood pressure), indicating limited incremental benefit from more complex modeling.

These findings suggest that high-risk patients can be identified preoperatively for closer monitoring or adjunctive therapy, whereas low-risk patients might safely receive routine follow-up. Prospective external validation is needed.

Although the overall discrimination of the multimodal model was moderate, the integration of echocardiographic and hemodynamic variables improved calibration and produced clearer separation of clinical risk strata. These parameters refined AI-ECG–based risk estimates, particularly for intermediate-probability cases, and provided mechanistic interpretability by linking electrical risk to atrial size and diastolic load. Thus, even with modest numerical gains in discrimination, the combined model adds clinical value as a framework for risk-adapted rhythm surveillance rather than as a standalone diagnostic tool. Its consistent performance across sensitivity analyses supports validity despite partial missingness in echocardiographic data. Quantitatively, the model’s 3-year Brier score (0.21) and ∼40-point gradient in 3-year arrhythmia-free survival between highest- and lowest-risk quartiles underscore strong calibration and clinically meaningful discrimination despite only moderate global C-index values.

### Temporal and missing-data sensitivity analyses and model robustness

Before interpreting temporal or modeling effects, it is important to clarify that recurrence was defined broadly, including both AF and AFl documented on any postblanking ECG regardless of antiarrhythmic drug use or repeat ablation. This inclusive definition, coupled with extended longitudinal follow-up and inclusion of concomitant surgical cases, likely explains the higher cumulative recurrence rate than contemporary reports that often measure 12-month freedom from atrial tachyarrhythmia lasting ≥30 seconds off antiarrhythmic therapy.

Given the 19-year study period encompassing major changes in surgical ablation practice, we evaluated model robustness through both temporal and missing-data sensitivity analyses. Although unadjusted recurrence rates differed among eras, inclusion of the surgical era in multivariable modeling did not meaningfully alter the performance or significance of principal predictors. This consistency supports that the ECG- and TTE-based predictors identified reflect patient substrate rather than procedural epoch, validating the approach of modeling all procedures within a unified predictive framework.

Because LA area measurements were missing in a significant portion of the cohort, a complete-case sensitivity analysis limited to 565 patients with available 2-dimensional data was also conducted. Results were directionally consistent with the multiply imputed model, with ECG-age and LA area remaining dominant predictors and overall discrimination unchanged (C-index 0.69 vs 0.65). The slightly stronger LA area association observed in the complete-case model (HR 1.031; 95% CI 1.015–1.047; *P* < .001) likely reflects regression dilution from mean-based imputation rather than bias. Importantly, missingness primarily resulted from the absence of standardized 2-dimensional measurements in earlier procedural eras rather than from patient-level disease severity, supporting a “missing at random” mechanism. Together, these analyses confirm that neither temporal variation nor incomplete data materially affected the study’s conclusions, reinforcing the robustness and generalizability of the identified predictors, particularly ECG-age and LA area, as determinants of long-term rhythm outcomes after surgical ablation.

### Echocardiographic predictors and atrial substrate

Consistent with previous studies, LA enlargement and diastolic dysfunction emerged as strong predictors of recurrence. This underscores the centrality of atrial myopathy in AF ablation failure. In surgical ablation cohorts, structural remodeling seems to be a central determinant of long-term outcomes. This aligns with previous studies, which found increased LA size was an independent predictor of AF recurrence after surgical ablation (whether Cox-Maze IV or thoracoscopic), regardless of lesion set.^2^ Similarly, Damiano et al^11^ demonstrated that LA size and function were critical determinants of success after Maze IV, particularly in patients with persistent AF and structural heart disease. Chelu et al^12^ also found that LA fibrosis on magnetic resonance imaging correlated with late AF recurrence after surgical ablation, reinforcing the role of substrate beyond just procedural technique. Our results support this and found that patients with larger and less compliant atria had higher recurrence rates. These findings support the concept that an adverse atrial substrate poses a significant challenge to surgical ablation, emphasizing the prognostic importance of atrial remodeling in this population.

Although AF subtype was strongly associated with recurrence on unadjusted analysis, it was excluded from multivariable modeling owing to inconsistent chart documentation and temporal variability in classification. Restricting the model to uniformly available, structured variables ensured analytic reproducibility and minimized misclassification bias. Nonetheless, the higher recurrence observed in patients with persistent AF aligns with previous reports and underscores the influence of chronic atrial remodeling on surgical ablation outcomes.

### AI-ECG biomarkers

The novel aspect of this study is the integration of AI-based ECG markers into risk prediction for surgical ablation outcomes. To the best of our knowledge, this is the first analysis to demonstrate the prognostic value of AI-ECG scores in a surgical ablation population. In particular, the AI-ECG-AF probability score proved to be a powerful predictor. Our results suggest that AI-ECG detects latent electrical features in a preoperative sinus ECG that suggest an atrial substrate predisposed to AF, which in turn significantly increases the risk of arrhythmia recurrence after surgical ablation. Similarly, the AI-derived ECG-age was an important predictor in our study and outperformed chronological age. Previous studies have demonstrated that excessive ECG-estimated age relative to actual age predicts AF recurrence after catheter ablation.^5^ In our multivariable analysis, only ECG-age (but not true age) remained significant, suggesting that ECG-age better captures the burden of diffuse cardiovascular aging and comorbidities that contribute to AF recurrence. Other AI-ECG disease scores, such as the HFpEF (diastolic dysfunction) score, were also associated with recurrence risk. These scores likely serve as proxies for underlying conditions that promote AF. For instance, the HFpEF ECG score correlates with elevated filling pressures,^7^ which can lead to LA dilatation and fibrosis. The significance of the HFpEF score and the mitral E-wave velocity in our model points to diastolic dysfunction as a contributor to surgical ablation failure.

### Clinical implications and future directions

Our study provides new insights into risk assessment for patients undergoing surgical AF/AFl ablation. It highlights that both echocardiographic markers of adverse atrial remodeling and AI-derived ECG biomarkers can be combined to predict long-term rhythm outcomes. From a practical standpoint, these findings suggest that patients with an adverse risk profile (eg, markedly enlarged LA, high AI-ECG-AF score) should be counseled about their higher likelihood of AF recurrence and monitored more closely after surgery. This should include closer follow-up ECG or ambulatory monitors for earlier detection of recurrent arrhythmia and thus facilitate prompt intervention to restore and maintain sinus rhythm. Conversely, patients identified as low-risk may be safely followed with routine care, potentially avoiding unnecessary interventions.

Our results also raise intriguing possibilities for preventative strategies. For instance, aggressive risk factor modification (strict blood pressure control, weight loss, treatment of sleep apnea, etc) might especially benefit those identified as high-risk by our model, to mitigate the substrate for recurrence. Previous trials in AF have shown that risk factor management can improve arrhythmia outcomes,^13^, ^14^, ^15^ and our identification of BMI and blood pressure as predictors reinforces the importance of addressing these factors. In addition, the modest gains of the RSF model suggest that even more sophisticated prediction tools could be developed, potentially incorporating biomarkers, proteomic or genetic data, or advanced imaging, to further refine risk stratification in the future. For example, future research could examine whether applying AI algorithms to echocardiographic data (ie, deriving novel AI-predicted echo features) further enhances predictive performance. This raises the potential for a truly multimodal model that seamlessly integrates AI-ECG and echo-derived predictors to improve risk stratification. Future models should also explore the dynamic changes in AI-ECG scores postoperatively, which could provide additional risk stratification insight.

### Study limitations

Our findings are best interpreted in light of several limitations. First, this was a retrospective analysis from a single integrated health care system that may limit generalizability. We attempted internal validation (including bootstrapping and cross-validation), but we lacked an independent external cohort to validate the models; thus, performance in other populations is unproven and should be assessed in future studies. Second, some clinically relevant variables, such as AF duration or more granular subtype distinctions (eg, long-standing persistent AF), were not uniformly documented and were thus excluded from the analysis, potentially confounding results. Similarly, details regarding the specific surgical ablation lesion sets were not consistently available, precluding analysis of their impact on recurrence. Postoperative antiarrhythmic drug usage was inconsistently documented, limiting our ability to assess its influence on arrhythmia recurrence. This limitation would likely bias the analysis toward underestimating recurrence, given that pharmacologic suppression could mask underlying substrate risk. Third, although the AI-ECG scores we used are validated for specific purposes, they were applied here in a new context (surgical AF/AFl ablation) that they were not explicitly trained for; their thresholds and performance characteristics in this scenario may not be fully optimized. Finally, our study was not designed to optimize or recalibrate the AI algorithms themselves; for example, the Cox model treated each AI-ECG score as a linear predictor and did not explore whether nonlinear effects (eg, an especially high ECG-AF score combined with a large LA might confer exponential risk) could improve prediction. The RSF analysis partially addresses this, but further work could refine how these scores are used in risk models. In addition, although the model demonstrated strong calibration across risk quartiles, indicating accurate estimation of average recurrence risk within groups, its moderate discrimination (C-index ∼0.67) highlights a key limitation: although it stratifies patients effectively at the population level, its ability to predict outcomes for individual patients remains limited, underscoring the need for further refinement in individualized risk assessment. Notably, the RSF model slightly outperformed the Cox model despite using the same top features, suggesting that nonlinear relationships or time-varying hazard effects may exist.

## Conclusion

Preoperative echocardiographic markers of adverse atrial remodeling and AI-ECG features were significant predictors of arrhythmia recurrence after surgical AF/AFl ablation. Preoperative AI-ECG biomarkers and TTE markers of atrial remodeling independently predicted AF/AFl recurrence, and integration of these metrics into a risk prediction model allowed stratification of patients into distinct risk groups.

## Declaration of generative AI and AI-assisted technologies in the writing process

During the preparation of this work, the authors used ChatGPT (OpenAI, version GPT-4) to assist with rewording sentences and proofreading to improve clarity, grammar, and readability. After using this tool, the authors reviewed and edited the content as needed and take full responsibility for the content of the publication.

## Disclosures

The authors have no conflicts of interest to disclose.
