# Supplementary material for: Transthoracic echocardiographic and artificial intelligence-enabled electrocardiography predictors of atrial arrhythmia recurrence after surgical ablation
Source: Heart Rhythm O2. 2025 Nov 7;7(1):9–17. doi: 10.1016/j.hroo.2025.11.001 (PMC12902217; doi:10.1016/j.hroo.2025.11.001)
Supplement: Supplemental Material [file mmc1.pdf]

Supplemental Material

Supplemental Table 1

Complete results of univariate analysis

Predictor	Hazard Ratio	95% CI Lower	95% CI Upper	p-value
ECG AF	3.912	3.258	4.696	1.950e-48
ECG Age	1.052	1.043	1.060	6.582e-35
Left Atrial End-Systolic Length by 2-D 4-Chamber View	1.056	1.045	1.067	1.312e-24
Left Atrial Area by 2-D Apical 4-Chamber View	1.052	1.042	1.063	2.768e-23
ECG HFpEF	1.301	1.232	1.375	3.58e-21
Left Atrial Area by 2-D Apical 2-Chamber View	1.057	1.045	1.070	2.48e-20
Age at Ablation	1.024	1.019	1.029	5.391e-20
Left Atrial Averaged End-Systolic Length by 2-D 4 and 2 Chamber	1.055	1.043	1.067	2.232e-19
Left Atrial End-Systolic 2-D Volume By A-L Apical 4 and 2-Chamber Method Using Averaged Length	1.008	1.006	1.010	7.295e-19
Left Atrial End-Systolic Length by 2-D 2-Chamber View	1.048	1.036	1.060	1.969e-16
Left Atrial End-Systolic 2-D Volume Index By A-L Apical 4 and 2-Chamber Method Using Averaged Length	1.016	1.012	1.019	6.532e-15
ECG AS	2.500	1.974	3.166	2.905e-14
Mitral Valve E to e` Ratio by PWD	1.018	1.014	1.023	3.335e-14
Mitral Valve E-Wave Peak Velocity by PWD	1.761	1.496	2.072	9.524e-12

Mitral Valve Lateral Annulus E to e` Ratio Diastolic Pulse Wave Doppler	1.03	1.021	1.039	1.205e-11
Diastolic Blood Pressure	1.015	1.009	1.021	1.398e-07
Left Ventricular Mass by M-Mode	1.005	1.003	1.007	3.44e-07
Mitral Valve Lateral Annulus Systolic Velocity by TDI	0	0	0.001	6.698e-07
ECG Low EF	1.727	1.361	2.191	6.888e-06
Left Ventricular Mass Index by M-Mode	1.009	1.005	1.014	9.123e-06
Interventricular Septum Diastolic Thickness by M-Mode	1.105	1.057	1.155	9.296e-06
Aortic Valve Systolic Peak Velocity by CWD	1.136	1.07	1.205	2.812e-05
Body Mass Index	1.026	1.013	1.038	3.058e-05
Pulmonary Vein Systolic Peak Velocity by PWD	0.177	0.078	0.402	3.451e-05
Aortic Valve Systolic Area by TVI	0.868	0.812	0.928	3.567e-05
Left Ventricular Posterior Wall Diastolic Thickness by 2-D	1.084	1.042	1.128	6.584e-05
Pulmonary Valve Systolic Peak Velocity by CWD	0.673	0.552	0.82	8.692e-05
Left Ventricular Internal End Diastolic Dimension by M-Mode	1.038	1.018	1.057	0.0001122
Aortic Valve Systolic TVI by CWD	1.004	1.002	1.007	0.0001766
Mid Ascending Aortic Diameter by 2-D	1.023	1.011	1.035	0.0002047
Left Ventricular Posterior Wall Diastolic Thickness by M-Mode	1.18	1.081	1.288	0.0002241

Left Ventricular Internal Systolic Dimension by M-Mode	1.046	1.021	1.071	0.0002442
Mitral Valve Medial Annulus e` Velocity by TDI	0.004	0	0.073	0.0002545
Pulmonary Valve Systolic Maximal Instantaneous Gradient by CWD	0.976	0.963	0.989	0.0004014
Mitral Valve Lateral Annulus a` Velocity by TDI	0	0	0.013	0.0005044
Aortic Valve Systolic Area by Velocity	0.892	0.836	0.951	0.0005127
Mitral Valve Lateral Annulus e` Velocity by TDI	0.016	0.001	0.171	0.000637
Left Ventricular EF Modified Quinones by 2-D	0.987	0.98	0.995	0.000805
Left Ventricular Mass by 2-D	1.002	1.001	1.003	0.0008393
Mitral Valve Medial Annulus Systolic Velocity by TDI	0	0	0.028	0.000921
Aortic Valve Systolic Area Index by Velocity	0.432	0.262	0.713	0.001027
Heart Rate	1.005	1.002	1.008	0.001047
ECG Male	1.361	1.127	1.642	0.001322
Aortic Valve Systolic Area Index by TVI	0.476	0.299	0.758	0.001756
Systolic Blood Pressure	1.005	1.002	1.008	0.002546
Left Ventricular EF Modified Quinones with Apical Correction Factor by 2-D	0.987	0.979	0.996	0.00261
Left Ventricular Mass Index by 2-D	1.003	1.001	1.005	0.004006
Mitral Valve Regurgitant Systolic Flow by PISA Method	0.998	0.997	0.999	0.004652

Mitral Valve Regurgitant Systolic Radius by PISA Method	0.502	0.302	0.835	0.007932
Left Ventricular Internal Systolic Dimension by 2-D	1.014	1.004	1.025	0.008631
Body Surface Area	1.4	1.077	1.82	0.01184
Mitral Valve Systolic ERO by PISA Method	0.501	0.293	0.859	0.01189
Left Ventricular Systolic Stroke Volume Index by Quantitative Doppler	0.992	0.986	0.998	0.01216
Left Atrial Maximum Volume by 2-D Method of Disks Biplane	1.003	1.001	1.006	0.01802
Left Ventricle Outflow Tract to Aortic Valve Time Velocity Integral Ratio Systolic	0.658	0.465	0.932	0.01828
Left Ventricular End Diastolic Volume MOD by 2-D 2-Chamber View	0.997	0.995	1	0.02128
Mitral Valve Systolic Regurgitant Volume by PISA Method	0.995	0.991	0.999	0.02271
Left Ventricular End Diastolic Volume Index 2-D Method of Disks Biplane	0.992	0.985	0.999	0.02371
Pulmonary Valve Regurgitant Late Diastolic Peak Velocity by CWD	1.284	1.028	1.605	0.0277
Tricuspid Valve Lateral Annulus Systolic Velocity by TDI	1.165	1.014	1.338	0.03078
Pulmonary Vein Atrial Contraction Reversal Peak Velocity by PWD	0.087	0.009	0.832	0.03404

Left Ventricular End Diastolic Volume MOD by 2-D Biplane Apical Views	0.997	0.995	1	0.03667
Estimated Right Atrial Pressure	1.018	1.001	1.036	0.03689
Left Atrial Maximum Volume by 2-D Method of Disks Four Chamber	1.003	1	1.005	0.03787
Heart Rate at Left Ventricular Stroke Volume Acquisition	1.005	1	1.01	0.03995
Tricuspid Valve Annulus Diameter Mean Normal Value	2.244	1.034	4.869	0.04092
Tricuspid Valve Annulus Systolic Excursion by M-Mode	0.974	0.949	0.999	0.04229
Left Atrial Maximum Volume by 2-D Method of Disks Two Chamber	1.003	1	1.005	0.04386
Left Ventricular Stroke Volume Index MOD by 2-D Apical Views	0.986	0.973	1	0.044
Right Ventricular End Diastolic Length by 2-D 4-Chamber View	0.982	0.964	1	0.04451
PA End Diastolic Pressure (CWD)	1.009	1	1.019	0.04491
Left Atrium Maximum Volume Index by 2-D Method of Discs Biplane	1.005	1	1.011	0.0453
Sino-tubular Junction Diameter by 2-D	1.023	1	1.047	0.05044
Mitral Valve Deceleration Time by PWD	0.998	0.997	1	0.0514
Mid Ascending Aorta Diameter 2-D, Upper Limit of Normal	1.102	0.998	1.216	0.05389
Interventricular Septum Diastolic Thickness by 2-D	1.021	0.999	1.044	0.05623

Mitral Stenosis TVI by CWD	0.99	0.979	1	0.05775
Left Ventricular EF MOD by 2-D 4-Chamber View	0.99	0.98	1.001	0.06287
Heart Rate for Z-Score	0.983	0.966	1.001	0.06956
Right Ventricular Outflow Tract Systolic Peak Velocity by PWD	0.433	0.17	1.104	0.07956
Descending Aortic Systolic Maximal Instantaneous Gradient by CWD	1.055	0.993	1.121	0.08265
Left Ventricular End Diastolic Volume MOD by 2-D 4-Chamber Apical View	0.998	0.996	1	0.08976
Left Ventricular Stroke Volume MOD by 2-D Apical Views	0.995	0.989	1.001	0.0927
Pulmonary Vein Diastolic Peak Velocity by PWD	1.652	0.911	2.993	0.09819
Aortic Valve Systolic Mean Gradient by CWD	1.004	0.999	1.009	0.1141
Descending Aorta Systolic Peak Velocity by CWD	1.863	0.855	4.061	0.1175
Aortic Arch Diameter by 2-D	1.028	0.992	1.066	0.1291
Diastolic Blood Pressure for Z-score	1.022	0.994	1.05	0.1322
Mitral Valve E to A Ratio at Baseline by PWD	1.101	0.963	1.259	0.1598
Left Ventricular End Systolic Volume MOD by 2-D 2-Chamber View	0.997	0.994	1.001	0.168
Left Ventricular Outflow Tract Systolic Mean Gradient by PWD	1.075	0.969	1.193	0.1704

Mitral Valve Regurgitant Systolic TVI by CWD	1.002	0.999	1.005	0.1707
Body Surface Area by Haycock Long Form	1.727	0.774	3.854	0.1824
Left Ventricular Internal End Diastolic Dimension by 2-D	1.007	0.997	1.016	0.1825
Sinus of Valsalva Diameter by 2-D	1.011	0.995	1.028	0.1846
PV Late Diastolic MIG (CWD)	1.007	0.996	1.018	0.197
Left Atrium Stroke Volume by Quantitative Doppler	0.997	0.993	1.002	0.2065
Right Ventricular Systolic Pressure by CWD	1.003	0.998	1.008	0.2066
Left Ventricular EF MOD by 2-D Biplane Apical Views	0.993	0.982	1.004	0.2075
Sinus of Valsalva Diameter 2-D, Upper Limit of Normal	1.065	0.963	1.178	0.2208
Left Ventricular Relative Diastolic Wall Thickness Percent by 2D by 2Times PW Method	1.007	0.996	1.018	0.2276
Mitral Valve A-Wave Peak Velocity by PWD	1.31	0.843	2.037	0.2295
Left Ventricle Outflow Tract to Aortic Valve Velocity Ratio Systolic	0.831	0.609	1.133	0.2425
Mitral Valve Annulus Diastolic Diameter by 2-D	0.829	0.603	1.141	0.2498
Left Ventricular Wall Motion Score Index by 2-D	1.281	0.834	1.966	0.2582
Left Ventricular EF MOD by 2-D 2-Chamber View	0.994	0.984	1.005	0.2655

Left Ventricular Cardiac Output by Quantitative Doppler	1.024	0.981	1.068	0.2781
Aortic Valve Systolic Maximal Instantaneous Gradient by CWD	1.002	0.998	1.007	0.2992
Mitral Valve Diastolic Mean Gradient by CWD	0.982	0.947	1.017	0.3049
Proximal Ascending Aortic Diameter by 2-D	1.012	0.99	1.034	0.3079
Left Ventricular End Systolic Volume MOD by 2-D Biplane Apical Views	0.998	0.994	1.002	0.3108
Systolic Blood Pressure for Z-score	1.01	0.991	1.029	0.3133
Right Ventricular Myocardial Performance Index	1.807	0.569	5.737	0.3152
Mitral Wave E Wave Velocity by CWD	0.838	0.59	1.191	0.3255
Left Ventricular Cardiac Index by Quantitative Doppler	0.958	0.872	1.052	0.3681
O2 Saturation	0.976	0.925	1.03	0.3791
Tricuspid Valve Lateral Annulus e` Velocity by TDI	4.231	0.164	109.402	0.3847
Corrected Aortic Valve Systolic Maximal Instantaneous Gradient	1.002	0.997	1.007	0.4233
Midazolam	0.991	0.968	1.014	0.4283
Left Ventricular EF Modified Quinones by M-Mode	0.992	0.973	1.012	0.435
Tricuspid Valve Regurgitant Systolic Peak Velocity by CWD	1.054	0.918	1.21	0.4547
Corrected Aortic Valve Systolic Mean Gradient	0.997	0.988	1.006	0.4602

Right Ventricular Outflow Tract Systolic TVI by PWD	1.018	0.969	1.07	0.4714
Left Ventricular End Systolic Volume MOD by 2-D 4-Chamber View	0.999	0.996	1.002	0.4912
Heart Rate at Mitral Valve Mean Gradient Acquisition	1.003	0.995	1.011	0.4929
Calculated Pulmonary Artery Capacitance	0.962	0.854	1.082	0.5164
Left Ventricular Stroke Volume by Quantitative Doppler	0.999	0.997	1.002	0.5409
Left Ventricular Outflow Tract Systolic TVI by PWD	0.996	0.981	1.01	0.5463
Left Ventricular Outflow Tract Systolic Peak Velocity by PWD	0.908	0.658	1.254	0.5591
% Normally Contracting Myocardium	0.998	0.992	1.004	0.5731
Mitral Valve Systolic Regurgitant Volume by Continuity Equation	0.998	0.993	1.004	0.5836
Mitral Regurgitant Systolic Peak Velocity by CWD	0.964	0.837	1.109	0.6059
Right Ventricular Ejection Time by PWD	0.999	0.995	1.003	0.6274
Aortic Annulus Systolic Diameter by 2-D	1.011	0.968	1.055	0.6279
Lidocaine Ointment Dose	0.799	0.318	2.008	0.6326
Tricuspid Valve Lateral Annulus a` Velocity by TDI	2.724	0.025	294.443	0.6748
Right Ventricular Internal Diastolic	1.004	0.985	1.023	0.6857

Basal Dimension by 2-D 4-Chamber View				
Tricuspid Valve Systolic Regurgitant Maximal Instantaneous Gradient by CWD	1.001	0.996	1.007	0.6858
IV Sedation Duration	0.996	0.978	1.015	0.7014
Heart Rate at Aortic Valve Mean Gradient Acquisition	0.998	0.987	1.009	0.7093
Tricuspid Valve Closure to Opening Time by CWD	1.001	0.997	1.004	0.723
Probe Time	0.997	0.983	1.013	0.7437
Mitral Valve Systolic ERO by Continuity Equation	0.882	0.396	1.965	0.7578
Left Ventricular Outflow Tract Systolic Diameter by 2-D	1.039	0.808	1.335	0.7663
Mitral Valve Annulus DiastolicTVI by PWD	0.994	0.95	1.041	0.8077
O2 Liters Per Min	1.015	0.835	1.233	0.8837
Left Ventricular EF Modified Quinones with Correction Factor by M-Mode	0.999	0.977	1.02	0.9016
Mitral Valve Medial Annulus a` Velocity by TDI	1.012	0.81	1.266	0.9138
Mitral Valve Systolic Regurgitant % by Continuity Equation	1	0.983	1.018	0.9792
ECG HCM	0.997	0.766	1.297	0.98
Fentanyl Dose	1	0.996	1.004	0.9836
Mitral Valve Regurgitant Systolic Aliasing Velocity by PISA Method	1	0.983	1.017	0.997
Right Ventricular Internal Diastolic Dimension by 2-D 4-Chamber View	1	0.977	1.023	1

Supplemental Table 2: Sensitivity analysis by surgical era (2006–2025)

Multivariable Cox proportional hazards model including Era 2 (2012–2017) and Era 3 (2018–2025) as covariates demonstrated nonsignificant trends toward lower recurrence risk compared with Era 1 (2006–2011). Inclusion of era did not materially alter the strength or direction of principal predictors, confirming model robustness across temporal cohorts.

**Variable**	**p-value (original)**	**p (sensitivity analysis)**	**Hazard Ratio (original)**	**HR (sensitivity analysis)**
**ECG AF**	<0.001	0.002	1.009	1.002
**ECG Age (per year)**	<0.001	<0.001	1.020	1.236
**ECG AS**	0.278	0.066	1.002	1.001
**ECG HFpEF**	0.076	0.036	1.068	1.084
**ECG Low EF**	0.300	0.537	0.998	1.000
**Diastolic Blood Pressure (per mmHg)**	0.010	0.003	1.008	1.103
**Body Mass Index (per kg/m²)**	0.036	0.092	1.013	1.059
**LA Area by Apical 4-Chamber View (per cm²)**	0.046	0.071	1.009	1.091

**Age at Ablation (per year)**	0.152	0.009	1.005	1.111
**Mitral E-Wave Peak Velocity (per 0.1 m/s)**	0.185	0.116	1.163	1.075
**Pulmonary Valve Systolic Peak Velocity**	0.721	0.646	1.032	0.980
**LV End-Diastolic Internal Diameter (per mm)**	0.754	0.644	1.002	1.023
**Heart Rate at SV Acquisition (per bpm)**	0.780	0.150	1.001	1.054
**LVEF (per %)**	0.190	0.494	0.995	0.959
**LV Mass (per gram)**	0.968	0.516	1.000	1.027
**Era_2**		0.062		1.118
**Era_3**		0.088		1.132

** Hazard ratios for ECG-AF, ECG-AS, and ECG-Low EF are expressed per 0.01-unit (1%) increase in the model output to enhance interpretability. The ECG-HFpEF score is reported per 1-unit increase on its native 0–3 scale. All other covariates are presented per their original measurement units.*

Supplemental Table 3: Complete-case sensitivity analysis limited to patients with available left atrial (LA) area measurements (n = 565)

Comparison of multivariable Cox model results with the multiply imputed main model demonstrates consistent direction and magnitude of key predictors, supporting robustness of findings despite missing echocardiographic data.

**Variable**	**p-value (original)**	**p (sensitivity)**	**Hazard Ratio (original)**	**HR (sensitivity)**
**ECG AF**	<0.001	0.027	1.009	1.004
**ECG Age (per year)**	<0.001	<0.001	1.020	1.036
**ECG AS**	0.278	0.794	1.002	1.001
**ECG HFpEF**	0.076	0.225	1.068	1.066
**ECG Low EF**	0.300	0.602	0.998	1.001
**Diastolic Blood Pressure (per mmHg)**	0.010	0.239	1.008	1.007
**Body Mass Index (per kg/m²)**	0.036	0.033	1.013	1.023
**LA Area by Apical 4-Chamber View (per cm²)**	0.046	<0.001	1.009	1.031

**Age at Ablation (per year)**	0.152	0.365	1.005	1.005
**Mitral E-Wave Peak Velocity (per 0.1 m/s)**	0.185	0.331	1.163	1.183
**Pulmonary Valve Systolic Peak Velocity**	0.721	0.860	1.032	0.978
**LV End-Diastolic Internal Diameter (per mm)**	0.754	0.992	1.002	1.000
**Heart Rate at SV Acquisition (per bpm)**	0.780	0.209	1.001	1.005
**LVEF (per %)**	0.190	0.427	0.995	0.994
**LV Mass (per gram)**	0.968	0.872	1.000	1.000

* Hazard ratios for ECG-AF, ECG-AS, and ECG-Low EF are expressed per 0.01-unit (1%) increase in the model output to enhance interpretability. The ECG-HFpEF score is reported per 1-unit increase on its native 0–3 scale. All other covariates are presented per their original measurement units.
